# Monoaminoxidase inhibitors as a cause of serotonin syndrome – a systematic case review based on meta-analytic principles

**DOI:** 10.1192/j.eurpsy.2021.968

**Published:** 2021-08-13

**Authors:** P. Truedson, M. Ott, H. Wikström, M. Maripuu, K. Lindmark, U. Werneke

**Affiliations:** 1 Sunderby Research Unit, Umeå University, Deparment of clinical science, division of psychiatry, Luleå, Sweden; 2 Department Of Public Health And Clinical Medicine – Medicine, Umeå University, Umeå, Sweden; 3 Department Of Clinical Sciences- Psychiatry, Umeå University, Umeå, Sweden; 4 Department Of Public Health And Clinical Medicine- Medicine, Umeå University, Umeå, Sweden

**Keywords:** Serotonin syndrome, Serotonin toxicity

## Abstract

**Introduction:**

Serotonin syndrome (SS) is a toxic state characterized by increased serotonin activity. It has been suggested that severe serotonin syndrome usually involves monoaminoxidase inhibitors (MAOIs).

**Objectives:**

To quantify in how far severe SS is associated with MAOIs.

**Methods:**

Systematic review and quantitative analysis of all SS cases published between 1 January 2004 and 31 December 2014. Severe SS was defined as cases, either requiring intensive care or resulting in death. Cases were included if they met the diagnostic criteria for SS according to at least one of the three diagnostic criteria systems (Hunter, Radomski and Sternbach).

**Results:**

Of the 299 included cases, 118 (39%) met the definition for severe SS. Eight cases had insufficient information to enable severity classification. Of the severe cases, 48 (40%) involved a MAOI. Of these, 67% related to psychiatric MAOIs, such as phenelzine and moclobemide and 33% to a somatic MAOI, such as methylene blue and linezolid. Of the remaining 173 non-severe SS cases, 24 cases (13%) involved a MAOI. In these, 12% related to a psychiatric MAOI and 83% to a somatic MAOI. One case (4%) had a combination of both. The odds ratio for MAOI involvement in severe versus non-severe serotonin syndrome was 4.3 (CI 2.4 – 7.5; p < 0.001).

**Conclusions:**

In the majority of published case reports, drugs other than MAOIs are involved in serotonin syndrome, even in severe cases. MAOIs are, however, more common in severe serotonin syndrome than in non-severe cases.

**Conflict of interest:**

M. Ott: scientific advisory board member of Astra Zeneca, Sweden. U. Werneke: received funding for educational activities on behalf of Norrbotten Region; Astra Zeneca, Eli Lilly, Janssen, Novartis, Otsuka/Lundbeck, Servier, Shire, Sunovion. Others: None

